# Cancer Clinicians’ Views Regarding an App That Helps Patients With Cancer Meet Their Information Needs: Qualitative Interview Study

**DOI:** 10.2196/23671

**Published:** 2021-05-06

**Authors:** Rebecca Richards, Paul Kinnersley, Kate Brain, Fiona Wood

**Affiliations:** 1 MRC Epidemiology Unit University of Cambridge Cambridge United Kingdom; 2 Centre for Medical Education Cardiff University Cardiff United Kingdom; 3 Division of Population Medicine Cardiff University Cardiff United Kingdom

**Keywords:** education, medical, medical information exchange, smartphone, mobile apps, mobile phone

## Abstract

**Background:**

Many patients with cancer have unmet information needs during the course of the illness. Smart devices, such as smartphones and tablet computers, provide an opportunity to deliver information to patients remotely. We aim to develop an app intervention to help patients with cancer meet their illness-related information needs in noninpatient settings. In addition to the in-depth exploration of the issues faced by the target users of a potential intervention, it is important to gain an understanding of the context in which the intervention will be used and the potential influences on its adoption. As such, understanding the views of clinicians is key to the successful implementation of this type of app in practice. Additionally, clinicians have an awareness of their patients’ needs and can provide further insight into the type of app and features that might be most beneficial.

**Objective:**

This study aims to explore cancer clinicians’ views on this type of intervention and whether they would support the use of an app in cancer care. Specifically, the perceived acceptability of an app used in consultations, useful app features, the potential benefits and disadvantages of an app, and barriers to app use were explored.

**Methods:**

A total of 20 qualitative, semistructured interviews were conducted with 22 clinicians from urological, colorectal, breast, or gynecological cancer clinics across 2 hospitals in South Wales. The interviews were audio recorded, transcribed, and analyzed using thematic analysis.

**Results:**

Clinicians felt that it would be acceptable for patients to use such an app in noninpatient settings, including during consultations. The benefits of this type of app were anticipated to be a more informed patient, an increased sense of control for patients, better doctor-patient communication, and a more efficient and effective consultation. In contrast, an increase in clinicians’ workload and poorer communication in consultations, which depended on the included app features, were identified as potential disadvantages. The anticipated barriers to app use included patients’ age and prior experience with smart technology, their access to smart devices, the confidentiality of information, and an avoidant coping approach to their condition.

**Conclusions:**

This study suggests that clinicians should support their patients in using an app to help them meet their information needs both at home and during consultations. This study highlights some of the potential barriers for this type of intervention in practice, which could be minimized during the intervention design process.

## Introduction

Most patients with cancer now largely manage their condition at home with less regular supervision by clinicians, which requires them to take a more active role in their treatment and survivorship [1,2]. To become a more activated patient and to manage the changes in daily life that come with cancer, patients require relevant and accurate information [3], and patients generally want as much information as possible about their condition [4]. However, recent studies conducted in Europe and the United States over the last 5 years have reported high rates of unmet information needs among patients with cancer [5-7]. In addition to limiting patients’ ability to participate in their care, unmet information needs are also associated with a lower quality of life, the loss of control over one’s life, increased anxiety and depression, and dissatisfaction with care [5,8-11].

Smart technology, including smartphones and tablet computers, has the potential to support the shift in cancer care to community settings and help patients meet their information needs by facilitating the delivery of information-based interventions to patients at home. However, a recent systematic review of the use of mobile devices to support patients with cancer with their information needs identified that available mobile interventions are mainly limited to helping patients with their treatment- or symptom-related information needs [12]. The authors concluded that more comprehensive interventions are required for patients managing the wider aspects of their condition in noninpatient settings.

This paper reports part of a series of studies documenting the systematic development of an app to help patients with cancer to meet their illness-related information needs [12,13], which followed the Medical Research Council (MRC) framework for the development of complex interventions in health care [14] and the person-based approach to enhance the acceptability and feasibility of such interventions [15]. A systematic review of the use of mobile devices to help patients with cancer meet their information needs reported that the vast majority of interventions aimed to improve the monitoring and management of treatment-related symptoms [12]. There were no interventions designed to meet patients’ full range of cancer-related information needs; more comprehensive interventions are required for patients to meet their information needs when managing their condition in noninpatient settings. Qualitative interviews were then conducted with a sample of patients with cancer to explore their views and preferences for a potential app to help them meet their illness-related information needs [13]. Suggestions for app features indicated the need for an app that supports patients to gather the key information that they need from their clinicians during time-constrained consultations, facilitates understanding, collates large amounts of information regarding available services, and helps patients navigate them. The anticipated benefits of this type of app included a more informed patient, improved quality of life, reduced anxiety, and increased confidence to participate in their care, which appeared to outweigh the potential disadvantages, such as potentially increased anxiety and distraction in consultations. Finally, patients anticipated that potential barriers to app use could be previous experience with smart technology, access to smart devices and the internet, an avoidant coping approach to their condition, and concerns about security and confidentiality of personal information.

In addition to an in-depth exploration of the issues faced by the target users of a potential intervention, it is important to gain an understanding of the context in which it will be used and the potential influences on the intervention [15,16]. As patients with cancer desire an app that would facilitate information gathering, exchange, and understanding during and between consultations with their clinicians [13], it is important to explore clinicians’ perceptions of the acceptability of this type of app to provide an opportunity to identify and minimize the potential barriers to its implementation in a clinical context [16,17]. In addition, clinicians have a potential role in encouraging the uptake of an app for patients with cancer following a diagnosis, as patients value the opinions of their clinicians and trust them as a source of reliable information [18,19]. Therefore, the support of clinicians will be key to the successful implementation of such interventions in practice [16].

The primary aim of the study reported in this paper is to explore clinicians’ views on the acceptability of an app for patients with cancer, including whether clinicians would support the use of an app in cancer care. Views on information exchange in consultations, useful app features, and the potential benefits and disadvantages of, and barriers to, app use were also explored.

## Methods

### Overview

Semistructured interviews were conducted with cancer clinicians at their clinics between June 2014 and November 2014. Participants were interviewed for this study before a qualitative interview study was conducted with a sample of patients with cancer [13]. Given that patients with cancer still report unmet information needs in recent years [5-7], it is prudent to continue with the development of interventions to support them and publish data that will help to build the evidence base in this field. National Health Service (NHS) ethical approval and R&D approval were granted (approval number: 14/WA/0066). Semistructured interviews were chosen because they enable a more personal and in-depth response from individuals compared with quantitative methods [20]. This method also allows participants the freedom to raise other relevant issues [19].

### Participants

We aimed to recruit a varied sample of clinicians to enable divergent views to emerge [20]. Cancer clinicians were recruited from colorectal, urological, breast, and gynecological cancer clinics within the University Hospital Wales and Velindre Hospital (a specialist cancer hospital) in South Wales, United Kingdom. These 4 cancer clinics were chosen because they have a variety of clinicians who deal with some of the most common cancers [21]. A decision was made to include a varied sample, including consultant surgeons, consultant oncologists, cancer nurse specialists (CNSs), and trainee clinicians (both medical and nursing).

### Recruitment

Clinical leads were identified and contacted so that the lead author (RR) could attend multidisciplinary team (MDT) meetings at each of the cancer clinics to present the study and invite clinicians to participate. Interested clinicians were emailed an information pack containing an invitation letter, information sheet, and reply form. It was not possible to attend an MDT meeting in all cancer clinics. In these circumstances, the lead clinician was asked to email their colleagues to invite clinical colleagues to participate in the study and to contact RR if they were interested. As a result of this recruitment method, the response rate could not be determined.

### Procedure

Face-to-face interviews were conducted with interested participants at the clinicians’ place of work. The interview was treated confidently, and only the research group had access to anonymized data. Clinicians provided written consent at the time of the interview and completed a demographic questionnaire that allowed us to describe the characteristics of our sample. Interviews were audio recorded, transcribed verbatim, and anonymized.

### Interview Topic Guide

Relevant literature informed the development of a semistructured interview topic guide [12]. The topics included information provision in consultations, experience with smart technology in consultations, perceived acceptability of an app intervention, perceived benefits and disadvantages of, barriers to app use, and useful app features for patients with cancer. At the beginning of the interviews, participants were told that an app could help with a wide range of things, such as patients’ information needs, communication in consultations, adherence to medication, and social support. Multimedia Appendix 1 provides a topic guide.

### Analysis

Participants were interviewed until the research team felt that data saturation was reached, sometimes referred to as the point of information redundancy. Although the concept of data saturation can be considered problematic in qualitative research [22], we considered this to have occurred when no new refinements to codes were made for at least three interviews. Data were managed using the qualitative analysis software package NVivo 10 (QSR International). Thematic analysis was selected to analyze interview transcripts, as this helps to provide insights by moving from a broad reading of the data to the conceptualization of codes and themes, followed by their interpretation [22]. The approach used was not considered purely inductive nor deductive but instead a blend of both approaches [22]. Each transcript was read several times to achieve familiarity by noting meanings and patterns. Initial codes were generated from each data item, and mind maps were created to identify the links between codes and possible overarching themes. Codes were then organized into meaningful subthemes and main overarching themes that captured the essence of the codes associated with them. Themes were reviewed and refined by reviewing each data item within a theme to ensure coherence.

RR, a doctoral student, collected and analyzed the data. A total of 5 transcripts were independently analyzed by a second author (FW) to allow collaborative discussion about the data and facilitate the interpretation of findings. Both authors maintained an awareness of how their personal characteristics and values may have influenced the data collection or analysis. For example, RR and FW are not medically trained and thus may not fully understand the clinical implications of the data. Participants knew that RR was also interviewing other cancer clinicians, possibly from the same clinic or hospital. Therefore, RR was aware of how this might have influenced participants’ trust and openness during the interviews and made every effort to build rapport and trust before the interview and to make the participants feel comfortable and at ease. RR assured participants that the interviews were confidential and that their views and opinions would not be discussed with other clinicians or their patients.

## Results

### Overview

In total, 20 interviews were conducted with 22 clinicians between June 2014 and November 2014. A total of 4 CNSs chose to be interviewed in pairs stating time constraints in the clinic; however, the remaining clinicians participated in individual interviews. The average length of the interviews was 27 minutes (range 20-39 min).

### Sample Characteristics

Participant characteristics are presented in Table 1. Of 22 clinicians, 12 (55%) were female and 10 (45%) were male. Overall, 36% (8/22) of the participants were CNSs, 23% (5/22) were consultant oncologists, 14% (3/22) were consultant surgeons, 23% (5/22) were trainee surgeon or oncologists, and 4% (1/22) were palliative care clinician. Of 22 clinicians, 7 (32%) were from urological cancer clinics, 6 (27%) were from colorectal cancer clinics, 5 (23%) were from gynecological cancer clinics, 3 (14%) were from breast cancer clinics, and 1 (4%) working in palliative care across subspecialities. All participants reported that they owned a smartphone or tablet computer.

**Table 1:** Sample characteristics.

ID (code)	Occupation	Cancer clinic
P1 (Onc^a^)	Oncologist	Gynecology
P2 (Onc)	Oncologist	Breast
P3 (Onc)	Oncologist	Breast
P4 (PCC^b^)	Palliative care clinician	All types
P5 (CNS^c^)	Cancer nurse specialist	Breast
P6 (TOnc^d^)	Trainee oncologist	Gynecology
P7 (CNS)	Cancer nurse specialist	Gynecology
P8 (CNS)	Cancer nurse specialist	Gynecology
P9 (CNS)	Cancer nurse specialist	Colorectal
P10 (CNS)	Cancer nurse specialist	Colorectal
P11 (Sur^e^)	Surgeon	Colorectal
P12 (Onc)	Oncologist	Colorectal
P13 (TSur^f^)	Trainee surgeon	Colorectal
P14 (Onc)	Oncologist	Gynecology
P15 (TSur)	Trainee surgeon	Urology
P16 (Sur)	Surgeon	Colorectal
P17 (TSur)	Trainee surgeon	Urology
P18 (CNS)	Cancer nurse specialist	Urology
P19 (Sur)	Surgeon	Urology
P20 (CNS)	Cancer nurse specialist	Urology
P21 (CNS)	Cancer nurse specialist	Urology
P22 (TSur)	Trainee surgeon	Urology

^a^Onc: oncologist.

^b^PCC: palliative care clinician.

^c^CNS: cancer nurse specialist.

^d^TOnc: trainee oncologist.

^e^Sur: surgeon.

^f^TSur: trainee surgeon.

### Interview Themes

From the interviews, 4 key themes were identified: (1) anticipated acceptability, (2) suggested app features, (3) anticipated benefits of app use, and (4) potential disadvantages or anticipated barriers to app use. Participants are identified with “P” followed by their identification number and the abbreviations of occupations listed in Table 1 (eg, P1 [Onc] is Participant 1, oncologist).

#### Theme 1: Anticipated Acceptability

Most clinicians reported that they do not currently use smart technology with their patients in consultations; however, 2 clinicians used apps to assist in explaining a patient’s condition to them. Most clinicians anticipated that it would be acceptable for patients to use a cancer app in consultations, reporting that patients already bring printed information or written question lists and that some use their smartphones to make notes during consultations:

Patients bring bits of paper, articles, all sorts of things. I mean, I think the patient population is changing...it’s just a screen with information on it really isn’t it? So I think, you know, the delivery is not critical...patients write things down quite a lot now. I think if patients did something on the app as opposed to the writing it down, I don’t think it makes any difference.

P19, surgeon

In contrast, 2 participants suggested that some older clinicians might perceive patients’ use of an app in consultations to be socially unacceptable and would resist the use of this type of technology in consultations. However, none of the senior clinicians in this study reported this to be an issue:

There are still, I’m sure the older clinicians...they might, they might have a big resistance to it.

P22, trainee surgeon

#### Theme 2: Suggested App Features

Clinicians suggested including the types of information most commonly requested by patients in consultations in an app for patients, such as information on the types of cancers and investigations, treatment options and side effects, cancer symptoms, recovery, and potential long-term effects:

...Things like why the investigations have been carried out, why we need to carry out extra tests, information about treatments, possible side effects and what psychological support is out there...and probably information on how to look after yourself as well. I mean smoking cessation, diet, stuff like that. Because a lot of patients ask that.

P21, cancer nurse specialist

Clinicians suggested including links to credible cancer information websites to signpost patients to reliable information, as they were aware that patients can often struggle to find reliable information outside of consultations, particularly on the internet:

I think if the patients are getting good information, so you know if this app is directing them to the right websites and everything...lots of patients go on the Internet and Google breast cancer and you get millions of hits back and they don’t know what is good information and what is bad information, so I think if this [the app] is going to point them in the right direction, clinicians would be up for that totally.

P5, cancer nurse specialist

Some clinicians felt that an app could also help patients to organize their care and suggested linking the app to the calendar feature on a smart device to remind patients of upcoming appointments. A medication log for patients to record their medication was also suggested by some clinicians:

I mean, I really like the idea of prompts and the diary and reminders, I mean patients forget, so maybe a day in advance to just remind them and then it reduces our DNAs [Did Not Attend]. Or a week before, “Have you asked your boss for that time off? Have you booked transport?” Or something like that. You get text messages for your bank appointments don’t you? Why not for your cancer appointments?...So act maybe as a diary manager.

P13, trainee surgeon

Clinicians suggested a feature that could store contact details to enable patients to contact their clinicians quickly where required, as they explained that patients often forget their designated nurse or consultant or lose their contact details:

The name of the clinicians that are looking after them, half the time they can’t remember contact details for their clinicians. That would be really useful. Summary of, you know, this is your diagnosis, this is your consultant, this is the number, the name of the nurse specialist, this is the name of the stoma nurse, these are their contact details, these are their email addresses, this is the secretary’s number.

P13, trainee surgeon

Many clinicians discussed that patients forget to ask questions in consultations and that this can lead to unmet information needs. Therefore, a question prompt list (QPL) feature was suggested to remind patients to ask important questions during consultations:

Many patients come and say to us, at the initial the shock of the diagnosis, they can’t think about anything else. So if they can formulate some questions, they won’t forget to ask, and they can keep their smartphone in front of the consultation, and keep ticking the boxes. That um, that’ll be useful actually for them, so they don’t forget anything.

P15, trainee surgeon

Many clinicians reported that they often use anatomical diagrams or images in consultations to help patients understand the information they are given, such as diagrams showing the location of the cancer and how operations will be performed. As a result, clinicians suggested an app feature that includes anatomical diagrams and images that could be used by clinicians to facilitate communication of information to patients in consultations:

Having pictures really helps...trying to explain what we are trying to do in terms of the operation as well, sometimes having a diagram actually makes a difference. And there are some apps where you could then look at your staging pictorially, that might be helpful to include in an app.

P14, oncologist

Clinicians also suggested including app features that would increase patients’ awareness of, and access to, patient support, as they explained that clinicians often forget or do not have time to provide this type of information. Suggestions included contact numbers of cancer charities and information on psychological support, such as support groups:

Erm, relevant information on how to find help, you know how to get extra support like erm, like a forum...or group support...or MacMillan numbers, Tenovus Cancer Care numbers.

P18, cancer nurse specialist

Um, local support groups...as well as national groups. I think more of the supportive side that perhaps we...we can’t really spend a huge amount of time on. Because I think we’re quite good at treating the disease and talking about the scientific part of the disease but it’s the, like the supportive aspect that we can’t provide enough time for, that I think would be of greatest benefit to a patient.

P17, trainee surgeon

#### Theme 3: Anticipated Benefits of App Use

Clinicians anticipated several potential benefits of an app that would help patients meet their information needs. The most commonly anticipated benefit of an app was a more informed patient. Clinicians suggested that an app could provide patients with a better understanding of cancer before consultations, which would enable them to have a more detailed discussion. In turn, clinicians expected that a more informed patient might develop more questions to ask:

I think it would have benefits in that the patient would be more prepared and therefore understand more about their own disease before their consultations, which would help. It may be that they ask more questions as a result of it.

P21, cancer nurse specialist

A minority of clinicians also anticipated that patients’ increased knowledge could lead to an increased sense of control over their lives by being able to plan ahead, which, in turn, might reduce their anxiety:

What do you think the benefits would be in the long term for patients?

Interviewer

It just gives them more control, um I think when they feel more control that helps them because it’s their lack of control, their lack of being able to plan, things just happening around them, and at least if you know what’s happening, so many patients come in and say, “Even though you kind’ve given me bad news, I feel better leaving than I did coming because I know what’s happening and I know you’ve got a plan.”

P1, oncologist

Clinicians anticipated that this type of app could also improve communication between patients and clinicians during consultations. It was thought that a QPL feature could act as an agenda for the consultation and, in turn, facilitate a more structured discussion while encouraging patients to communicate their concerns:

The first steps I think you need to take are fairly simple and that’s things like frequently asked questions...The app can be introduced, obviously, at various different stages, but certainly prior to the second visit, if they download the app and they have been on to answer those, ask those questions,...common questions that are asked...frequently asked questions, they may want to go through those before they then come back and see you a second time, or even the first time.

P12, oncologist

Well I think clearer communication actually, knowing you’re following the patient’s agenda and what their problems are enables you to, you know, clarify things quicker, and to answer questions better.

P4, palliative care clinician

Clinicians suggested that this might improve the efficiency of the consultation and increase clinicians’ confidence that they have met the patients’ information needs:

Hopefully it could form a very clear structure for a consultation which, you know, means it’s probably more time efficient. Consultations can be quite long sometimes, particularly when you’re trying to get the complex situation across, so I think there are benefits in terms of time.

P11, surgeon

#### Theme 4: Potential Disadvantages of and Barriers to App Use

On the other hand, a minority of clinicians were concerned that an app for patients could potentially increase their workload and the length of consultations if it encourages patients to contact clinicians (via a contacts feature) or ask questions in consultations (via a QPL feature). However, clinicians believed that the many potential advantages of such an app would outweigh this potential disadvantage:

It could potentially slow down consultations, we have to bear that in mind. But I think in the end of you have a quality consultation, in the end it probably speeds things up overall. As well as improves the quality of that consultation.

P12, oncologist

A small number of clinicians were also concerned that an app might hinder communication during consultations by distracting patients, who may then miss information. Similarly, some clinicians felt that app use during consultations could potentially reduce patients’ nonverbal communication, which is used by clinicians to assess whether patients have understood the information:

If it doesn’t divert the consultation...because they are constantly looking at the app, and they won’t be able to listen to what we say, and they may even miss it. So I presume that’s the downside of it actually...I personally don’t like um, somebody sitting in front of me and they’re just on the smartphone ticking boxes, not listening to what I say, because a lot of it...face to face, eye contact on the person, and from the eye contact I can see whether the patient has understood it or not.

P15, trainee surgeon

Clinicians anticipated several potential barriers to the use of this type of app in practice. The main anticipated barriers were patients’ age and prior experience with smart technology, where many clinicians believed that many older patients lacked the knowledge and experience to be able to use, or want to use, an app. In addition, clinicians expected that some older patients might have problems with physically using an app because of poor eyesight and/or dexterity:

I think in general, and it is a vast generalization, cancer patients tend to be older patients and the older patients tend not to be able, quite so versed, in using apps and all that sort of stuff. So I think at the moment you might not get a great uptake. Give it ten years and I think yeah, I think everyone will be using it and the people who are in their sixties, seventies now, who are then going on to get cancer in their eighties and things...it’ll be very useful for.

P22, trainee surgeon

You have the very practical problems with patients of this age group because their eyesight is often poor, their dexterity might not be that good, you know on an iPhone rather than an iPad.

P13, trainee surgeon

Some clinicians were concerned that the cost of a smart device would be a barrier for some older patients who do not currently have access to one. However, clinicians expected that these patients could gain access to a device via their family or friends:

It would be sort of potentially be a barrier you know for the older ones who may not have the equipment or want the equipment but then again may have family members that would be willing.

P20, cancer nurse specialist

Some clinicians were concerned about the confidentiality of patients’ information on an app, particularly due to cancer being a sensitive topic:

I think storage of information, sensitive information is the main issue. If they have a smartphone or, you know, a tablet device that isn’t locked then potentially if you put sensitive information on it, it could be easy to view, so you might need to put a password onto the app.

P14, oncologist

Finally, some clinicians indicated that a minority of patients appear to have an avoidant coping approach to their illness and so do not wish to have extensive information. As such, clinicians anticipated that this type of patient would not want to use this type of app:

One thing I guess I would say is that you’re always going to get the patient that will do everything, and you’re always going to get the patient that will do nothing. There are those patients that will use everything and everything that they can access they will do...and others won’t, you know?

P5, cancer nurse specialist

## Discussion

### Principal Findings

To our knowledge, this is the first study to explore the views of cancer clinicians regarding the development of a novel app intervention to help patients with cancer to meet their illness-related information needs in noninpatient settings. The primary aim of this study is to understand clinicians’ views on the value of this type of intervention, the type of app that they anticipate to be most useful for patients, and whether clinicians would support the use of an app in clinical practice. Overall, clinicians felt it would be acceptable for patients to use such an app to support their information needs, including consultations. Clinicians’ awareness of the barriers to information exchange during, and outside of, consultations with patients were reflected in the type of app features they suggested. The benefits of this type of app were anticipated to be a more informed patient, an increased sense of control for patients, better doctor-patient communication, and a more efficient and effective consultation. In contrast, an increase in clinicians’ workload and poorer communication in consultations, which depended on the included app features, were identified as potential disadvantages, although clinicians believed that these would be outweighed by the benefits. The anticipated barriers to app use included patients’ age and prior experience with smart technology, access to smart devices, confidentiality of information, and an avoidant coping approach to their condition. Overall, the views of clinicians largely mirror the views of patients with cancer on this type of intervention [13].

Most clinicians reported that they had not previously used an app to assist them with patients in consultations; however, all clinicians owned a smart device and were familiar with this technology. This finding is likely because of the lack of availability of patient-facing apps that are reliable and developed by researchers or health organizations [23,24], as an increasing number of clinicians use apps for a wide variety of work-related tasks [25]. Importantly, clinicians appeared to be supportive of the development of an app to help patients meet their information needs. This finding is consistent with previous studies that reported clinicians’ positive perceptions and expectations for mobile interventions for other chronic health conditions [26-28].

Clinicians’ suggestions for app features reflected their awareness of barriers to information exchange during and outside of consultations with patients with cancer. First, clinicians suggested app features that would help patients to better self-manage their condition by providing detailed information about their cancer. This type of information might help prevent unnecessary hospitalizations [29]. Clinicians also suggested links to reliable websites to help patients source accurate information. As the internet is now a common health information resource, studies have highlighted the importance of guiding patients to filter accurate health information [30,31]. This could help avoid patients becoming unnecessarily anxious and prolonging consultations with their clinicians, leaving room for more informed discussions. Clinicians also suggested additional app features that were not thought of by patients themselves in our previous qualitative study [13], including a feature to help them organize their care, such as appointment reminders, a medication log, and a feature to store clinicians’ contact details.

Second, clinicians suggested app features to enable patients with cancer to overcome barriers to communication in consultations, such as a QPL to help patients remember to ask important questions. Clinicians felt that this type of feature would help patients to make their information needs clear to the clinician, instead of passively relying on the clinician to relay information. It is important for patients to voice their concerns and provide relevant information for their clinicians in order for clinicians to formulate the correct diagnosis and prescribe or amend treatment for patients [32]. Clinicians also suggested a feature to assist *them* in imparting information to patients more effectively using diagrams or images; however, this feature might be better placed in a clinician-facing, rather than patient-facing, app.

Third, clinicians felt that an app could help with raising awareness of, and signposting patients to, cancer support services, such as contact numbers for cancer charities or information on support groups, which they felt would be beneficial for patients. This finding is supported by previous studies on the benefits of social support during cancer [33]. Clinicians explained that they were not often able to impart this information because of limited time in consultations; thus, this presents an example of how technology can help to relieve pressure on the NHS services and help to meet information needs of patients with cancer.

The most commonly anticipated outcome of this type of intervention was a more informed patient, which is consistent with patient expectations [13]. Clinicians further highlighted the benefits that they themselves might receive as a result of using a patient-facing app, including a more structured and efficient consultation. However, although some previous studies on the use of paper-based QPLs in cancer consultations have reported a decrease in consultation length, the evidence is generally mixed [34,35]. Indeed, some clinicians were concerned that this type of app could lead to an increased workload if the app increased patient contact and question-asking. Some previous studies on clinicians’ perceptions of their involvement in mobile symptom-monitoring interventions for patients with cancer have reported that increased workloads and technical issues were problematic in clinical practice [17,36,37]. However, these interventions were used equally by clinicians and patients, whereas a patient-facing app that is used independently of the clinician would limit the potential impact on clinicians’ workloads. In addition, clinicians in this study believed that the advantage of an improved consultation might outweigh the potential increase in workload. Subsequently, studies of digital and paper-based patient-facing interventions that are used *during* allocated consultation times have been found to be acceptable by clinicians [38,39].

A number of clinicians in our study were concerned that an app might hinder communication during consultations by distracting patients and that some older clinicians might be particularly resistant to this change in consultations. These findings are unsurprising, as previous studies have reported that some clinicians perceive the use of a smartphone in clinical settings to be unprofessional because of the association of mobile phones with poor quality social contact [40,41]. However, as stated earlier, previous studies have shown that digital interventions that are used by patients in consultations are acceptable by clinicians in practice [38,39]. In addition, senior clinicians were interviewed for this study and found the idea of an app for patients with cancer to be acceptable.

Other potential barriers to app use identified by clinicians included patients’ age and experience with smart technology, access to smart technology and the internet, and confidentiality of patients’ medical information, which were also concerns of patients with cancer [13] and clinicians of previous studies of mobile interventions for chronic health conditions [26-28]. However, clinicians recognized that patients’ age and prior experience with smart technology is only a temporary potential barrier and an app that would not require the input of sensitive information might circumvent concerns of confidentiality.

### Implications

This study presents novel findings on the views of cancer clinicians regarding the development of an app for patients with cancer, including the potential outcomes and benefits of this type of intervention. In line with the MRC framework [14] and person-based approach [15] for the development of complex interventions in health care, these findings can be used, in combination with the findings from our patient study [13], to develop intervention objectives and inform the selection of app features. For example, based on clinicians’ views reported in this study, and in support of patients’ views, the objectives of the intervention might be to facilitate the development of patients’ understanding and self-management of their condition, and it is anticipated that this could be achieved by including app features that enable patients to (1) better self-manage their condition by sourcing accurate information outside of consultations and improving the organization of their care through the use of reminders and logs, (2) overcome barriers to communication in consultations by encouraging question-asking and participation in their care, and (3) identify and access cancer support services that will provide further information and support where needed (such as psychological support). This study identified the potential benefits of a patient-facing app for the clinicians themselves and the potential disadvantages of, and barriers to, this type of app. These findings can be considered during app design to optimize its uptake, usability, and usefulness [15]. For example, clinicians were concerned that patients could be distracted in consultations, so an objective would be to design an app that will only be referred to briefly in consultations but not actively used throughout. Similarly, to circumvent some clinicians’ concerns about the confidentiality of information, a further objective would be to design an app that does not require the input of sensitive information.

### Limitations

A varied sample of clinicians, including a variety of roles, settings, patient types, and career lengths as well as a balance of both genders, is a strength of this study. However, there were several limitations to consider. It was not possible to calculate the response rate for this study nor to collect the key characteristics of those who declined to participate. In addition, all clinicians were smart technology owners. Therefore, the sample may not be representative of the general population of clinicians and may have included those with more favorable perceptions of an app than those who chose not to participate. However, statistics suggest that ownership of smart technology among clinicians is common, where up to 90% of health care professionals own a smart device, and new technologies will continue to be integrated into health care services [42,43]. Joint interviews with 4 clinicians may have prevented these participants from discussing important issues that they might have talked about in a separate interview; however, most interviews were conducted individually at length.

Providing examples of types of app features that could be used by patients with cancer before beginning the interview might have influenced some participants’ responses because of social desirability. The risk of this bias was minimized as the interviewer explained that all opinions were valued, both positive and negative, to develop an app that would be most useful for future patients.

Finally, participants were asked to reflect on a hypothetical scenario in which an app could be available for patients in the future. Participants were also asked to anticipate *the potential* benefits and disadvantages of, and barriers to, a hypothetical app. As a result, the data are not necessarily grounded in concrete experience and therefore may not translate into engagement.

### Conclusions

To our knowledge, this study is the first to explore the views of cancer clinicians regarding an app that aims to help patients with cancer meet their illness-related information needs in noninpatient settings. Clinicians appear to be supportive of the development of an app and its use in consultations and suggested the types of app features that they anticipate to be useful for patients; specifically, an app that would enable patients to better self-manage their condition, overcome barriers to communication in consultations, and identify and access cancer support services. The potential outcomes of this type of intervention were highlighted, including the benefits for both the patients and clinicians, and the potential benefits of this type of intervention appeared to outweigh clinicians’ few minor concerns.

## Appendix

Multimedia Appendix 1 Clinician topic guide.

## Abbreviations

CNS: cancer nurse specialist
MDT: multidisciplinary team
MRC: Medical Research Council
NHS: National Health Service
QPL: question prompt list
