# Supplementary material for: Cancer Clinicians’ Views Regarding an App That Helps Patients With Cancer Meet Their Information Needs: Qualitative Interview Study
Source: JMIR Cancer. 2021 May 6;7(2):e23671. doi: 10.2196/23671 (PMC8138703; doi:10.2196/23671)
Supplement: Multimedia Appendix 1 [file cancer_v7i2e23671_app1.docx]

**Clinician topic guide**

Prologue

1. Introduce myself, explain where I am from, and ensure they’re comfortable.

2. Check understanding of reason for meeting, give an opportunity for questions:

**“Before we start, I wonder if you have any questions about this study or about why I’ve come to talk with you today?”**

Set the focus of the interview and explain the app. Base this around the following script:

**“Thank you for agreeing to take part in this study. I want to understand what clinicians of cancer patients think of a potential smartphone/tablet app for patients with cancer. The interview will take around half an hour. This app would be for use *after* diagnosis and would potentially help patients with their information needs (e.g. by providing information within the app and links to external websites, etc) and help patients to communicate with their clinicians in consultations (e.g. by bringing a prepared list of questions to consultations). The app could also help with things like adherence to medications (e.g. audio medication prompts) and social support (e.g. links to support groups, telephone helplines). At the end of this study, we aim to develop an app based on the information gathered from interviews with clinicians, cancer patients and their relatives. I am interested in your views on a potential smartphone/tablet app for cancer patients. There are no right or wrong answers to my questions, I am interested in what *you* think.”**

3. After establishing what is understood about the study, and answering any questions, explain that the interview will be recorded: **“I would like to record what you say as that saves me having to scribble when you’re talking and means that I can concentrate on what you’re saying. The recording will only be heard by people who are working on this project. The interview will be transcribed and your identity and the identity of any person you talk about today will be anonymised in any published work. Is that okay with you?”**

4. Obtain consent for the interview and for the recording. If not already done, set up and switch on the recording equipment while the volunteer signs the consent form.

5. Explain how the interview will work: “**I’ve planned some ideas about the sorts of things I’d like us to talk about today, and if it’s okay with you we’ll try and base our conversation around those points. Having said that, if you want to tell me about anything that I don’t ask about, please just tell me. Also, if you find a question difficult to answer, please say and we can move on or I could try to ask it in a different way. Of course, if you’d prefer not to answer any question, that is absolutely fine. There aren’t any right or wrong answers to anything I ask you, we’re just interested in your own opinions and experiences. Does that all sound alright to you?”**

6. Obtain demographic information.

**Opening question**

Can you tell me why you were interested in taking part in this study?

**Information provision**

*Rationale: establish amount and source of information provision*

How do you decide how much information you give to patients about their illness?

How do you provide information to patients about their illness? (prompt: verbally, written information, combination]

Do you recommend any sources of information for patients and family/friends? (Prompt: websites, helplines, and charities. What sources?)

**Communication in consultations**

*Rationale: barriers to doctor-patient communication in consultations*

What kinds of issues do you think clinicians are faced with when talking to patients about their illness?

Do you think there are any barriers to communicating with patients in consultations?

**Experience with Smart technology**

*Rationale: to establish clinician’s personal/professional experience with mobile technology and apps*

What experience do you have of using a smartphone/tablet computer?

What do you use your smartphone/tablet computer for?

Are you familiar with ‘apps’?

Do you currently use any apps for work-related purposes? Why/why not?

**Perceived ease of use and acceptability**

*Rationale: to anticipate ease of use and clinician’s support and acceptability of patient app use*

Do you think patients and their relatives would want to use it?

Do you think patients and their relatives would find this kind of app easy to use?

Do you think clinicians would be happy for patients to use this kind of app during their consultations?

Do you think clinicians would be happy for patients to use this kind of app at home?

**Perceived benefits of app**

*Rationale: perceived benefits of an app for cancer patients*

What benefits do you think there might be for *patients* using the app?

What kinds of benefits do you think there might be for *clinicians?*

Do you think families or friends of patients would want to use this kind of app? Why?

Do you think the app would have an impact on communication with your patients? How would it affect communication?

Do you think the app would have an impact on how patients and their family and friends talk about their illness and share information about their illness?

**Perceived barriers of the app**

*Rationale: perceived barriers of app*

Do you think there may be problems with patients using this app? What do you think the problems might be?

**Training needs**

Due to the app, do you feel that clinicians would require training in how to deal with more active patients/changing communication?

**Desired app features**

*Rationale: establish what clinicians would like a cancer app to do/not do*

What sort of app features do you think would be most useful for patients?

What sort of app features do you think would be most useful for relatives/friends?

Is there anything that you would want the app to do? If so, why?

Is there anything that you would not want the app to do? If so, why?

**Patient type**

*Rationale: anticipate what type of patient might find an app most useful*

Are there any particular types of patient that you think might find this mobile technology most useful? (prompt: age groups, patients at different stages of diseases). Why?
